# *ZLL/AGO10* maintains shoot meristem stem cells during *Arabidopsis* embryogenesis by down-regulating ARF2-mediated auxin response

**DOI:** 10.1186/s12915-015-0180-y

**Published:** 2015-09-10

**Authors:** Farshad Roodbarkelari, Fei Du, Elisabeth Truernit, Thomas Laux

**Affiliations:** BIOSS Centre for Biological Signaling Studies, Faculty of Biology, Albert-Ludwigs-Universität, 79104 Freiburg, Germany; Present address: ETH Zürich LFW E 51, Universitätstr. 2, 8092 Zürich, Switzerland

**Keywords:** ARF2, ARGONAUTE10, Stem cell identity

## Abstract

**Background:**

The shoot meristem gives rise to new organs throughout a plant’s life by the activity of pluripotent stem cells in the meristem center. Organ initiation at the periphery of the shoot meristem is triggered by the accumulation of the phytohormone auxin at the initiation site. Loss-of-function mutants of the *ZWILLE/ARGONAUTE10/PINHEAD* (*ZLL/AGO10/PNH*) gene terminate shoot meristem stem cells late in embryogenesis and can form a leaf or a leaf-like structure instead, indicating that *AGO10* activity is required to maintain shoot meristem stem cells undifferentiated.

**Results:**

Here, we addressed whether stem cell maintenance by *AGO10* involves regulation of auxin. We found that in *zll-1* mutants, auxin accumulation and expression of the response reporter *DR5:GFP* are elevated, and transcription of the *Auxin Response Factor 2 (ARF2)* gene is upregulated. Downregulation of *ARF2* significantly restores stem cells in *zll-1* mutants, whereas increased expression of *ARF2* enhances differentiation of stem cells in *zll-1* mutants. We further found that upregulation of the *AGO10* effector gene *REVOLUTA* restores *ARF2* expression and stem cell maintenance in *zll-1* embryos.

**Conclusions:**

Our results indicate that maintenance of shoot meristem stem cells by *AGO10* involves negative regulation of auxin signaling and, via REV-mediated downregulation of *ARF2* expression, auxin response.

**Electronic supplementary material:**

The online version of this article (doi:10.1186/s12915-015-0180-y) contains supplementary material, which is available to authorized users.

## Background

All above-ground plant organs originate from the shoot apical meristem, which is established during embryogenesis [1, 2]. Within the shoot meristem, the central zone contains three layers of slowly dividing pluripotent stem cells, which respectively give rise to the epidermis, subepidermal cells, and internal tissues. Stem cell daughters enter differentiation pathways in the surrounding peripheral zone, where lateral organs are initiated, and in the underlying rib zone, where the pith of the shoot is formed [3].

Several key regulators of stem cell maintenance have been identified in the past, including a feedback loop between stem cells and underlying niche cells, named the organizing center [4]; regulation of cytokinin synthesis and response [5]; and localized expression of miRNAs [6]. The *ARGONAUTE10* (*AGO10*) gene is required for stem cell maintenance during embryogenesis and for initiation of axillary meristems during postembryonic development. Loss of *AGO10* function in the Landsberg *erecta* (L*er*) accession results in stem cells differentiating into a flat apex, leaf-like structures, or normal-appearing leaves [7, 8]. Expression of the stem cell marker *CLAVATA 3* (*CLV3*) is correctly initiated at the transition embryo stage of the *zll-1* mutant, but is discontinued at the bent-cotyledon embryo stage [9, 10]. *AGO10* encodes a member of the AGO protein family that binds small RNAs and acts as effectors of RNA interference in plants and animals [11, 12]. In contrast to canonical AGO proteins, however, binding of miR165/166 to AGO10 does not result in degradation of the target mRNAs, encoding HDZIP III transcription factors, but in their increase. Therefore, a function of AGO10 as a decoy AGO protein, which limits loading of miR165/166 onto AGO1 and thus reduces degradation of *HDZIP III* mRNAs, has been proposed [13].

One of the earliest events in organ initiation by the shoot meristem is the accumulation of auxin via directional cell-to-cell transport [14–16]. Auxin can trigger the degradation of AUX/IAA repressor proteins and consequently the activation of AUXIN RESPONSE FACTORS (ARFs) transcription factors [17, 18]. The *Arabidopsis* genome contains 23 ARFs [19], specific subsets of which regulate a multitude of developmental processes [20–23]. The stem cell-containing central domain of the meristem appears relatively insensitive to auxin [24] and displays increased mechanical stiffness; these factors have been suggested to limit organ formation [25].

The initiation of differentiated organs in the place of the stem cells in *zll* mutants raises the question whether AGO10 might negatively regulate auxin function. To address this question, we analyzed the interplay between *AGO10* and auxin during embryogenesis. Our results show that *AGO10* downregulates both auxin signaling and auxin response, including expression of *ARF2*. Downregulation of *ARF2* levels in *zll-1* embryos via an artificial miRNA (amiR) recovers stem cell maintenance. Furthermore, increased *REV* activity in *zll-1* restores *ARF2* repression and stem cell maintenance. Together, these results provide a framework of how AGO10 maintains shoot meristem stem cells by negatively regulating auxin signaling.

## Results

### Auxin signaling and response are increased in *zll-1* embryos

**Fig. 1:**
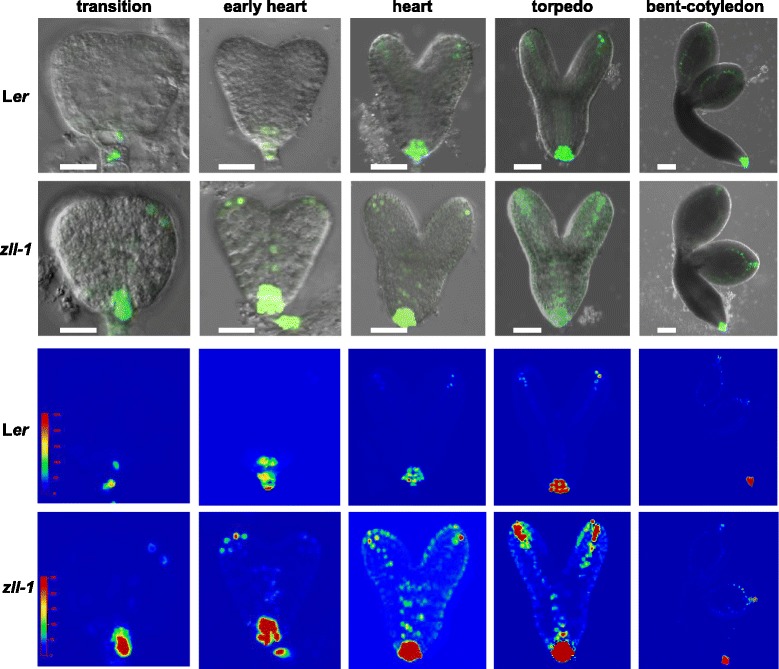
Auxin response is enhanced in *zll-1* mutant embryos. *Upper two lanes* show overlays of *DR5:GFP* signal and differential interference contrast images. Images were taken at the same settings. In the *bottom two lanes*, the corresponding heat maps of DR5:GFP expression are shown*.* Rainbow gradient from *blue* (no expression) to *red* (high expression) is used. Scale bars: 15 μm, except for bent-cotyledon stage, 30 μm. Genotypes and embryo stages are indicated

To address whether auxin regulation might be altered in *zll-1* mutants, we first monitored auxin response with the *pDR5:nlsGFP* reporter (Fig. 1, Additional file 1: Figure S1, Additional file 2: Table S1). In wild-type embryogenesis, *pDR5:GFP* expression was detected from the globular stage onwards at the root pole and additionally from the heart stage onwards in the tips and developing vasculature of the cotyledons. In *zll-1* embryos, *pDR5:GFP* expression levels were increased in all regions where expression was found in wild type. Additional expression was detected in the vasculature of the embryo axis of early heart to torpedo stage embryos and in the epidermis of cotyledonary tips of transition to heart stage embryos.

To investigate what causes the increased *DR5:GFP* response in *zll-1*, we first monitored expression of the tandem auxin signaling sensor R2D2 [26], where the *pRPS5a:DII-n3xVenus* (*DII-Venus*) signal is inversely correlated to auxin signaling and where the *pRPS5a:mDII-ntdTomato* (*mDII-ntdTomato*) signal acts as transcription control. In addition, a *pCLV3:er-CFP* transgene was used to monitor shoot meristem stem cells.

**Fig. 2:**
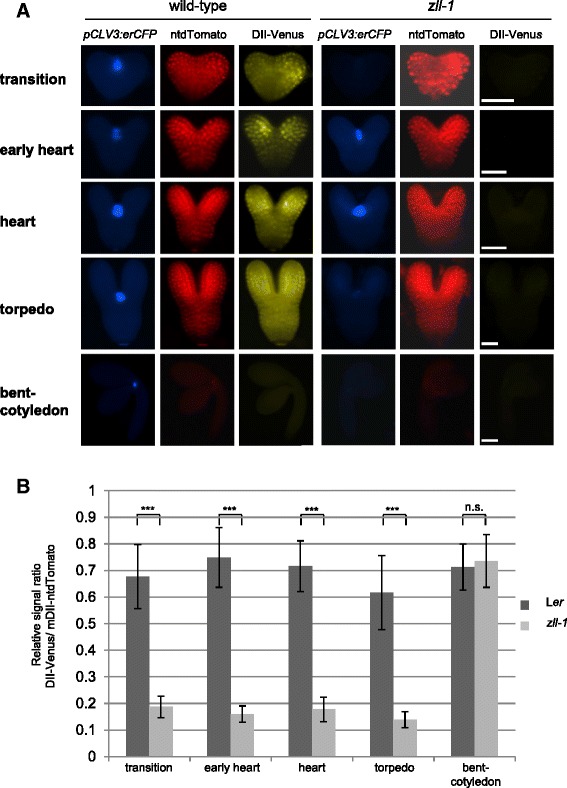
Auxin signaling is elevated in *zll-1* embryos. **a** Expression of the stem cell markers *pCLV3:erCFP*, *pRPS5a:mDII-ntdTomato* (*ntdTomato*), and *pRPS5a:DII-n3xVenus* (*DII-Venus*) in embryos at the indicated stages and genotypes. The erCFP signal was recorded with 1,000 ms exposure time, ntdTomato with 400 ms, and DII-Venus with 3,000 ms for all samples. The CFP signal is shown in *blue*, DII-Venus in *yellow*, and ntdTomato in *red*. Scale bars: 10 μm for transition to heart stages, 30 μm for torpedo and bent-cotyledon stages. **b** DII-Venus to ntdTomato signal ratios during embryogenesis. ***p <0.001; *n.s.* not significant

In wild-type transition to torpedo stage embryos, expression of the *pRPS5a:mDII-ntdTomato* reporter was detectable in the cotyledons and at a lower level in the shoot apex and the basal part (Fig. 2). In bent-cotyledon stage embryos, the mDII-ntdTomato signal was close to background level in the majority of the analyzed embryos (Fig. 2 and Additional file 3: Table S2). This indicates that the *pRPS5a* driver is active in wild-type embryogenesis until the torpedo stage. Expression of the *pRPS5a:DII-n3xVenus* auxin signaling reporter was detectable in a similar pattern between the transition and the torpedo stages (Fig. 2 and Additional file 3: Table S2). As expected, no signal was detectable in bent-cotyledon stage embryos, where the driver is not active (Fig. 2).

In the *zll-1* mutant, the expression of *pRPS5a:mDII-ntdTomato* did not show any detectable difference compared to wild type (Fig. 2), demonstrating that the *pRPS5a* driver works as in wild type. In contrast to wild type, however, the DII-Venus signal was not detectable in the majority of *zll-1* embryos from transition to torpedo stages (Fig. 2 and Additional file 3: Table S2), indicating increased auxin signaling. We notice that this embryo-wide downregulation of the DII-Venus reporter in *zll-1* embryos contrasted the localized upregulation of *DR5:GFP* expression (Fig. 1). This difference might be due to the reported higher sensitivity of DII-Venus compared to DR5 [27] and the different molecular requirements of the two auxin markers: DII-Venus relies on the presence of the TRANSPORT INHIBITOR RESPONSE1 (TIR1) F-box protein family and on the presence of auxin itself [28–30], and DR5 relies additionally on the presence of ARF transcription factors. One mechanistic interpretation of the different patterns of both reporters is that increased early signaling does not always result in ARF2-dependent transcriptional activities.

**Fig. 3:**
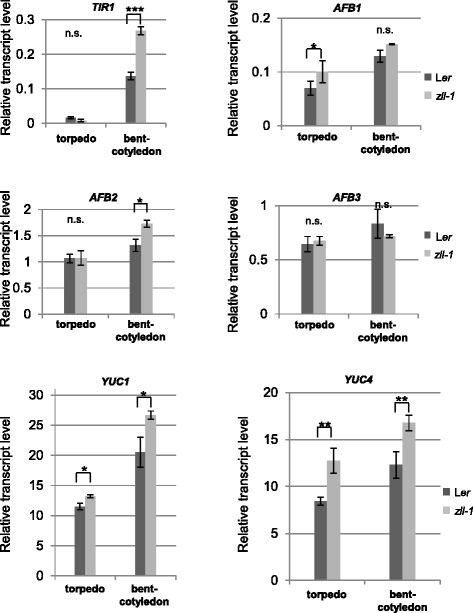
Auxin biosynthesis and receptor gene expression is upregulated in *zll-1* embryos. mRNA levels were determined by qRT-PCR of receptor genes (*TIR1, AFB1–3*) and biosynthesis genes (*YUC1, YUC2*) relative to the reference gene *At4g26410* and standard deviations of three biological replicates are shown. Student’s *t*-test was used to calculate *p*-values. *p < 0.05, **p < 0.01

*TIR1* and *AUXIN SIGNALING F-BOX 1–3* (*AFB1–3*) auxin receptor mRNAs are targets of miR393 [31, 32], which has been found to preferentially bind to AGO1 [33] and thus could be sensitive to the levels of AGO10. Notably, we found that *AFB1* mRNA levels were slightly increased compared to wild type in *zll-1* torpedo stage embryos and *TIR1* and *AFB2* mRNA levels in *zll-1* bent-cotyledon stage embryos (Fig. 3). Thus, the observed decrease in DII-Venus signal could be partially due to the increase of TIR/AFB expression.

In *Arabidopsis*, the main source of auxin is from tryptophan via two enzymatic steps, catalyzed by tryptophan amino transferases (TAA1, TAR1, and TAR2) [34, 35] and the YUCCA (YUC) family monooxygenases [36]. We found that the expression of *YUC1* and *YUC4* was weakly upregulated in the *zll-1* mutant compared to wild type (Fig. 3), whereas *TAA1* and *TAR2* expression was not significantly changed (Additional file 4: Figure S2).

In summary, these results indicate that between transition and torpedo stages, *zll-1* embryos display higher levels of auxin synthesis and signaling compared to wild type.

### *ARF2* levels in *zll-1* mediate stem cell termination

We next asked whether increased auxin signaling might contribute to the shoot meristem defects in *zll-1* mutants through *ARF* auxin effector genes. For an initial characterization we chose *ARF2* as a *TAS3*-regulated *ARF* gene [37], and *ARF6* as a miR167-regulated *ARF* gene [38], which are expressed during embryogenesis but not in the embryonic shoot apex [21]. Because no loss-of-function mutants were available in the L*er* ecotype, we downregulated *ARF2* and *ARF6* mRNA levels via artificial miRNAs (amiR). Changing *ARF6* expression had no effect on shoot meristem formation in wild type or in *zll-1* (Additional files 5, 6, 7, and 8: Tables S3–S6) and was not further pursued.

**Fig. 4:**
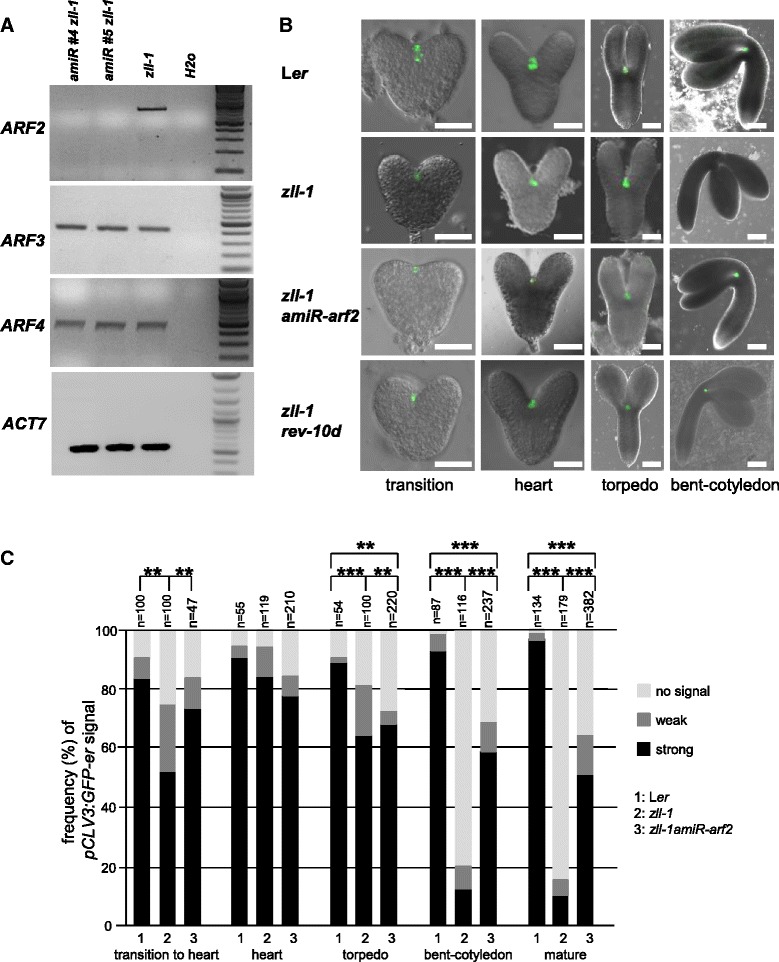
Stem cell maintenance in *zll-1* is restored by reduced *ARF2* and increased *REV* levels. **a** sqRT-PCR shows that *ARF2* transcript levels are strongly downregulated, whereas ARF3 and ARF4 transcripts are unchanged in two independent *p35S:amiR-arf2 zll-1* lines compared to untransformed *zll-1*. **b** Expression of the shoot meristem stem cell marker *pCLV3:GFP-er* (*green*) is discontinued in *zll-1* embryos at the bent-cotyledon stage, but recovered in *zll-1* expressing *p35S:amiR-arf2* and in *zll-1 rev-10d* double mutants. Scale bars: 20 μm. **c** Percentage of *pCLV3:GFP-er* expression levels in given genotypes during embryo development. *n* number of embryos analyzed. *p*-values were calculated by the Chi-square test: **p < 0.01, ***p < 0.001

Expression of *p35S:amiR-ARF2* strongly reduced *ARF2* mRNA levels detected by semi-quantitative reverse rranscriptase polymerase chain reaction (sqRT-PCR) in torpedo stage embryos (Fig. 4a), but did not have any effect on the shoot meristem development in wild type (not shown), in accordance with previous findings [23]. By contrast, in *zll-1* bent-cotyledon stage embryos, *p35S:amiR-ARF2* expression partially rescued stem cell maintenance as measured by the expression of the stem cell marker *pCLV3:GFP-er* (*p* < 0.001; Fig. 4b,c) and the presence of an active shoot meristem at the seedling stage (Additional file 9: Table S7).

**Fig. 5:**
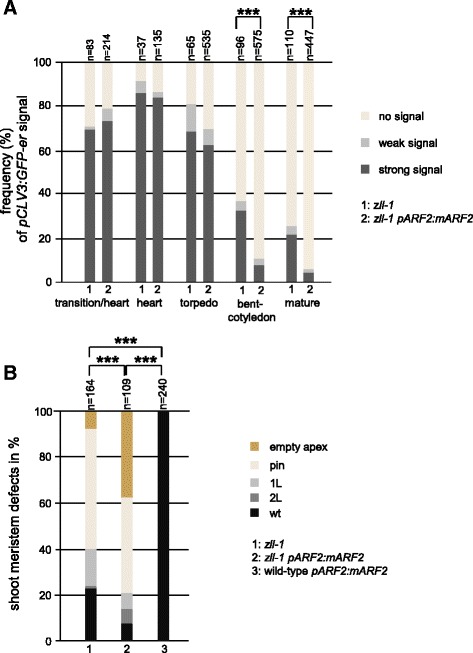
*zll-1* stem cell defects are enhanced by increased *ARF2* levels. **a** *pCLV3:GFP-er* expression is decreased in *zll-1 pARF2:mARF2* bent-cotyledon and mature embryos compared to *zll-1.* **b** The severity of shoot meristem defects is enhanced in 14-day-old seedling of *zll-1 pARF2:mARF2* compared to *zll-1*. Genotypes and embryo stages are indicated. The Chi-square test was used to calculate *p*-values. ***p < 0.001; *n* number of analyzed embryos. Shoot meristem defects: *empty apex* differentiated stem cell, no organ formation; *pin* single filamentous structure; *1 L* one central leaf; *2 L* termination into two leaves; *wt* wild-type-like

In the converse experiment, we upregulated *ARF2* mRNA levels by expressing a *TAS3*-resistant *pARF2:ARF2m* transgene in order to avoid degradation of *ARF2* mRNA via the *TAS3* siRNA. In the *zll-1* background, this resulted in a significant (*p* < 0.001) reduction of *pCLV3:GFP-er* expression in bent-cotyledon stage and mature embryos (Fig. 5a) and of shoot meristem maintenance (Fig. 5b, Additional file 10: Table S8). In addition, the severity of shoot meristem defects was enhanced (Fig. 5b, *p* < 0.001). By contrast, *pARF2:ARF2m* expression did not cause any obvious phenotypic change in the shoot apical meristem when expressed in the wild type (Fig. 5b). This suggests that ARF2 levels are not a limiting factor in shoot meristem maintenance in wild type, but are in *zll-1*, consistent with the increased auxin accumulation in *zll-1*.

Together these results suggest that downregulation of *ARF2* expression is required for shoot meristem stem cell maintenance during embryogenesis by *AGO10* activity.

### *REVOLUTA* mediates downregulation of *ARF2* expression by *AGO10*

**Fig. 6:**
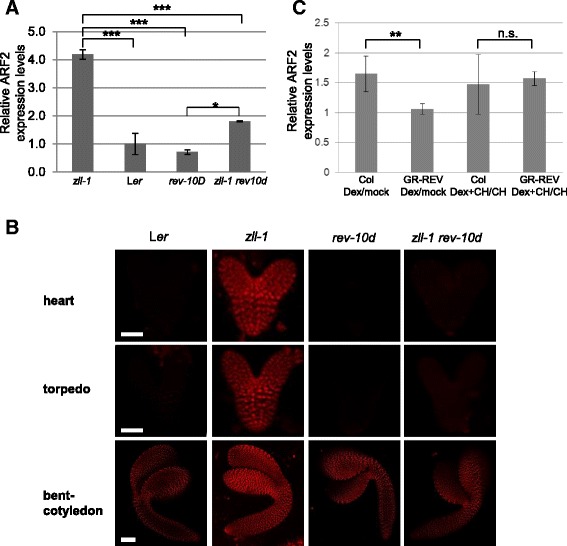
*ARF2* expression is negatively regulated by *AGO10* and *REV.* Quantitative real-time polymerase chain reaction experiments of three independent biological replicates with standard deviation. **a** *ARF2* expression levels in torpedo stage embryos of the indicated genotypes relative to wild-type levels. **b** *pARF2:ntdTomato* expression (*red*) is upregulated in *zll-1* heart to bent-cotyledon stage embryos compared to wild type (L*er*) and this upregulation is suppressed by the *rev-10d* mutation. Scale bars: 15 μm for heart and torpedo stage embryos and 30 μm for bent-cotyledon stage embryos. **c** *ARF2* expression levels in 5-day-old shoot apical meristems of the indicated genotypes and treatments. *Bars* represent the fold changes of *ARF2* in response to dexamethasone relative to mock treatment and in response to dexamethasone (*DEX*) + cycloheximide (*CH*) relative to CH-control in wild type (Col) and the DEX-inducible *35S::GR-REVd* line (*GR-REV*), respectively. Genotypes and treatments are indicated. Transcription levels are normalized to the reference gene *At4g26410*. Significance tested by Student’s *t*-test is indicated. *p < 0.05, **p < 0.01, ***p < 0.001; *n.s.* not significant

One possible reason why reduction of *ARF2* activity suppresses shoot meristem termination in *zll-1* embryos is that *AGO10* negatively regulates *ARF2* expression levels. Indeed, we found by qRT-PCR that *ARF2* mRNA levels were 3–4-fold increased in *zll-1* torpedo stage embryos compared to wild type, whereas ARF3 and ARF4 levels were not affected (*p* < 0.001; Fig. 6a, Additional file 11: Figure S3).

Next we analyzed expression of the transcriptional reporter *pARF2:nlstd-Tomato (pARF2:ntdTomato)* during embryogenesis. In wild type, *pARF2:ntdTomato* was hardly detectable from heart to torpedo stages and was detected throughout the embryo at the bent-cotyledon stage (Fig. 6b, Additional file 12: Figure S4). By contrast, in *zll-1* heart to bent-cotyledon stage embryos, *pARF2:ntdTomato* was uniformly expressed at higher levels compared to wild type (Fig. 6b, Additional file 12: Figure S4). We note that this expression pattern differs from the one reported in the Columbia accession [21], which could be due to different *ARF2* expression levels in the L*er* accession used in our experiments.

In summary, these data indicate that *AGO10* is required to downregulate *ARF2* transcription from heart to torpedo stages throughout the embryo.

AGO10 prevents accumulation of miR165/166 in the embryonic shoot meristem and thus promotes expression of their target *HD-ZIP III* genes [13]. We therefore asked whether HD-ZIP III activity might mediate repression of *ARF2* transcription via AGO10. To this end we introgressed the miR-resistant *rev-10d* mutation [39], which as a single mutant does not display any shoot meristem defect (not shown), into the *zll-1* mutant. This resulted in a significant rescue of shoot meristem formation in *zll-1* embryos (*p* < 0.001; Additional file 13: Figure S5A), including the expression of the stem cell marker *pCLV3:GFP-er* (*p* < 0.001; Fig. 4b, Additional file 13: Figure S5B).

qRT-PCR studies demonstrated that the *rev-10d* mutation largely suppressed the upregulation of *ARF2* mRNA levels in the *zll-1* mutant (Fig. 6a, *p <* 0.001), and that inducible *p35S:GR-REVd* activity repressed *ARF2* expression (Fig. 6c, *p* < 0.01). Addition of cycloheximide abolished the effect of REV on *ARF2* mRNA (Fig. 6c), indicating that it is indirect. Likewise, *rev-10d* suppressed the expression levels of the *ARF2* transcriptional reporter in *zll-1* embryos (Additional file 12: Figure S4).

Taken together, this indicates that *AGO10* represses *ARF2* transcription in the embryonic shoot meristem through REV activity.

## Discussion

During embryonic shoot meristem formation, the two basic functions of the meristem need to be established in spatially separated domains: stem cell maintenance in the meristem center, and organ initiation in the periphery [3, 40]. Several mutants of shoot meristem development result in an inactive stem cell pool, resulting in a terminated apex without the initiation of an organ [41–43]. By contrast, *zll* mutant embryos frequently form a leaf structure in place of the stem cell pool in the meristem center [8, 44]. This defect first becomes visible at the bent-cotyledon embryo stage when *zll-1* embryos are unable to maintain expression of the shoot meristem stem cell marker CLV3 [10]. In this study, we have addressed the underlying mechanisms of this phenotype and analyzed the role of auxin and *AGO10* interaction in stem cell maintenance.

### AGO10 negatively regulates auxin signaling and response during embryogenesis

Our results provide several lines of evidence that increased auxin function in *zll-1* contributes to the failure to maintain shoot meristem stem cells in the embryo, as detected by the loss of CLV3 expression and the ectopic organ formation in place of the stem cells. First, the perception capacities and auxin levels were elevated in the *zll-1* mutant throughout the embryo. Second, reduction of *ARF2* levels partially suppressed stem cell termination in *zll-1*, whereas increased *ARF2* levels increased stem cell defects.

In protoplast transfection assays, ARF2 acts as transcriptional repressor of *DR5:GUS* [45], which would suggest that upregulation of DR5:GFP in *zll-1* is not a direct response of elevated ARF2 levels and therefore other factors are probably involved. We consistently found that, in addition to *ARF2*, other components of auxin response, such as *TIR1/AFB* and *YUC* genes, were upregulated in *zll-1* mutants, albeit at weaker levels.

How is the upregulation of *ARF2* accomplished? Previous studies showed that AGO10 promotes expression of the transcription factor REV by blocking degradation of its mRNA via miR165/166 [13]. Our results show that, in turn, REV mediates downregulation of *ARF2* by *AGO10*, because increased *REV* levels in the *zll-1* mutant reduced *ARF2* expression and suppressed stem cell defects in the *zll-1* mutant. It is likely that *ARF2* repression by REV is not direct, because repression did not take place when protein synthesis was blocked. Consistent with this notion, REV was not found to bind to *ARF2* in a genome-wide map of REV binding sites [46]. Curiously, a previous study showed that overexpression of *REV* in seedlings directly activates expression of auxin biosynthesis genes of the tryptophan-dependent indole-3-pyruvic acid biosynthetic pathway, *TAA1* and *YUC5* [46]. In our studies, however, we found that *YUC1* and *YUC4* levels and possibly auxin accumulation were upregulated in *zll-1* embryos, suggesting that AGO10 affects auxin synthesis by a mechanism independent of REV, or that REV acts differentially on auxin synthesis during embryo and seedling development.

These findings indicate that AGO10 is required to reduce auxin activity in the embryo and that this process is essential for maintaining shoot meristem stem cells [14, 47].

Genetic interaction studies and expression experiments indicate that *AGO10* is required to potentiate WUS-mediated maintenance of *CLV3* expression in the embryonic shoot meristem stem cells [10]. In contrast to *WUS*, which is confined to the developing shoot meristem region, *AGO10* is expressed in a broader pattern, including the shoot meristem and the pro-vasculature [10]. However, localized expression of *AGO10* in the vasculature is sufficient for shoot meristem development, suggesting that *AGO10* can act non-cell autonomously. Consistent with the broad *AGO10* expression pattern in embryos, we found enhanced auxin signaling and response and increased *ARF* expression throughout the *zll-1* embryo. Because organ initiation in the shoot meristem requires the accumulation of auxin at the initiation site through directional transport from surrounding tissues [48], one interesting question to be answered in the future is whether stem cell loss in *zll-1* embryos is the consequence of enhanced auxin activity inside or outside the shoot meristem.

## Conclusion

In this study, we show that the ARGONAUTE family member AGO10 negatively regulates auxin signaling during embryogenesis. Consequently, the conversion of stem cells into an ectopic leaf-like organ in the *zll-1* mutant appears to be due to upregulated auxin activity involving increased transcription of the auxin response factor *ARF2*.

## Methods

*Arabidopsis* plants were grown as described previously [10]. The shoot apical meristem of L*er*, *zll-1*, and transgenic lines was analyzed 14 days after germination. Plant transformation was done by the *Agrobacterium*-mediated floral dipping method [49].

Wild-type and *35S::GR-REVd* seedlings were grown on Murashige and Skoog medium plates in long-day conditions at 22 °C for 4 days. Induction of GR-REVd was done as described previously [50].

### Constructs

Primers for three artificial miRNAs of *ARF2* were designed using the WDM3 online software and amiRNAs were amplified as described previously [51]. Specificity of amiR sequences for *ARF2* was confirmed using BlastN. The artificial miRNAs were subcloned into the *Bam*HI site of the *pEG287* vector. The *amiR-ARF2* with a 35S promoter upstream and a Nos-Terminator downstream were cloned in *Hind*III/*Eco*RI sites of a *pGreenII* binary vector. The promoter of *ARF2* was amplified using forward primer 5′-ACTAACTTGATGAATGAAAGAGTCGCAGCG-3′ and reverse primer 5′-ACTAAGCTTACCTTCCGAAGCTCAGATCTGTTTC-3′, and cloned into the *Hin*dIII restriction site of a modified *pGreenII* vector containing a ligation-independent cloning (LIC) tail [52]. The *ARF2* coding region was amplified using forward primer 5′-TAGTTGGAATAGGATTTCGTAGGATCCATGGCGAGTTCGGAGGTTTC-3′ and reverse primer 5′-AGTATGGAGTTGGATTTCGTTGGATCCTTAAGAGTTCCCAGCGCTG-3′, containing LIC tails.

The amplified *ARF2* coding region containing LIC tails was cloned into a modified *pGreenII* vector containing a LIC tail as described previously [52]. To generate a TAS3-resistant *ARF2* version, the *ARF2* coding region was cloned into a *pJet2.1* vector and mutated using the Stratagene site directed mutagenesis kit (cat. no. 200518, Stratagene, La Jolla, CA, USA) with forward primer 5′-GCAAGCGGACTTTCAAGGGTGCTCCAGGGACAGGAGTACTCGACCTTGAGGACGAAAC-3′ and reverse primer: 5′-GTTTCGTCCTCAAGGTCGAGTACTCCT GTCCCTGGAGCACCCTTGAAAGTCCGCTTGC-3′. The *ARF2* transcription marker sequence was amplified with forward primer 5′-TAGTTGGAATAGGATTTCGTAGGATCCATGGCGAGTTCGGAGGTTTC-3′ and reverse primer 5′-AGTATGGAGTTGGATTTCGTTGGATCCTTAAGAGTTCCCAGCGCTG-3′, and subcloned into a modified *pGreenII* vector containing a LIC tail containing the *ARF2* promoter.

### Microscopy

Embryos were dissected from maternal tissues and mounted in 10 % glycerol. They were analyzed with a Zeiss AxioImager. A1 microscope (Carl Zeiss Microscopy, Jena, Germany) or a Zeiss LSM 700 confocal laser scanning microscope (Carl Zeiss MircoImaging GmbH, Göttingen, Germany). The images were processed using Adobe Photoshop Elements 2.0.

The integrated signal intensity of each embryo was measured in a median plane and calculated using the ROI tool of Image J software. Analysis of the measured data and drawing of bar plots were done using Microsoft Excel software.

### Semi-quantitative PCR of *ARF2, ARF3*, and *ARF4*

To measure the efficiency of *amiR-ARF2*, RNA of *zll-1* and *zll-1amiR-ARF2* lines was extracted using a Qiagen RNeasy kit (Qiagen, Hilden, Germany). cDNA amplification was done with the Qiagen cDNA synthesis kit. Amplification of cDNA sequences were performed using forward primer 5′-TTCGATGCTTACCAGAGAAGGT-3′ and reverse primer 5′-TTGAGTCTGTCCCATTCATGTTG-3′ for *ARF2*; forward primer 5′- GATTCCAGAGGGTCTTGCAAGGTCAAGAAATTTTTCC −3′ and reverse primer 5′- CAACGCAGGGGACAGCCGTC-3′ for ARF3; and forward primer 5′-TCCCTCGGTTTCTCTCCCACACT-3′ and reverse primer 5′-AGCAAATTTCTTGACCTTGCAAGACCCTTGGAAACC-3′ for ARF4. As reference, *ACT7* (At5g09810) was amplified with forward primer 5′-GGTGAGGATATTCAGCCACTTGTCTG-3′ and reverse primer 5′-TGTGAGATCCCGACCCGCAAGATC-3′ [53].

### Quantitative PCR

Torpedo stage embryos were dissected out of the ovule, and RNA was extracted using the Qiagen RNeasy kit. cDNA was generated using the Invitrogen SuperScript III first-strand synthesis system for qPCR (cat. no. 11752–050; Life Technologies, Carlsbad, U.S.A).

qRT-PCRs were done using the Roche LightCycler 480 SYBR Green I kit (Cat. No. 04707516001; Roche Diagnostics GmbH, Mannhein, Germany), with forward primer 5′-TGCTGGTCCGCTTGTGACGG-3′ and reverse primer 5′-TGCCGCCTGGTTCGTCGAAG-3′ of *ARF2*; forward primer 5′-CACGGAGGTTCAGGCAGATGCA −3′ and reverse primer 5′-CAACGCAGGGGACAGCCGTC-3′ of *ARF3*; forward primer 5′-TCCCTCGGTTTCTCTCCCACACT-3′ and reverse primer 5′-ATGGGGTTTCCGGGTGGGGT-3′ of *ARF4*; forward primer 5′-CGAATATAACGCATATAACGCC-3′ and reverse primer 5′-CCATAAACATAGAGAGAGAGAGGTTC-3′ for *TAA1*; forward primer 5′-GCTCTTCACTGCTTCAAAGAGCAC-3′ and reverse primer 5′-TCTGTCTTTCACCAAAGCCCATCC-3′ for *TAR2*; forwards primer 5′-GAGAGACGAAATCAAGGGGT-3′ and reverse primer 5′-GAGGTAAAGACAAAACGAGAACTG-3′ for *YUC1*; and forward primer 5′-ATGGGCACTTGTAGAGAATCAG-3′ and reverse primer 5′-CGGACCAGGAACGAAGAT-3′ for *YUC4*. For TIR1, we used forwards primer 5′- TGAGGAAACTAGAGATAAGGGACTGC-3′ and reverse primer 5′- CACGGAACAAGAAGACATCCAAAGG-3′ for AFB1; forwards primer 5′-ACTTGTTGTCGGGCTGTGAGAG-3′ and reverse primer 5′-CTCTGGAGGATGTTCATCAATGACTTC-3′ for AFB2; forwards primer 5′-CAAGTATGAAACAATGCGATCCCTTTG-3′ and reverse primer 5′-TTCTTCCATCCGGTTATTATCATTCTCG-3′ for AFB3; and forwards primer 5′-AAGGAATGCTCTATGTGTTGAATGGATG-3′ and reverse primer 5′-AACCTTCTCTCTTTCATCTTCTTCATTCTG-3′. At4g26410 [54] was used as a reference gene, and amplified with forward primer 5′-GAGCTGAAGTGGCTTCCATGAC-3′ and reverse primer 5′-GGTCCGACATACCCATGATCC-3′.

## Additional files

Additional file 1: Figure S1. DR5:GFP signal is upregulated in *zll-1* embryos. Integrated fluorescence intensity of DR5:GFP signal of whole embryos at the indicated stages. *n* numbers of embryos analyzed, *n.s.* not significant; ****p* < 0.001. (PPT 107 kb) Additional file 2: Table S1. Increased auxin response in the *zll-1* mutant. (DOC 51 kb) Additional file 3: Table S2. DII-Venus levels are reduced in *zll-1* mutants. (DOC 65 kb) Additional file 4: Figure S2. Transcript levels of auxin biosynthesis genes *TAA1* and *TAR2. TAA1* (A) and *TAR2* (B) transcript levels are not significantly altered between *zll-1* and L*er* wild-type embryos. qRT-PCR transcript levels relative to reference gene *At4g26410* and standard deviation of three biological replicates are shown. Student’s *t*-test was used to calculate *p*-values. (PPT 112 kb) Additional file 5: Table S3. Increased *ARF6* expression does not affect shoot apical meristem development in *zll-1. (DOC 43 kb)*
Additional file 6: Table S4. Increased *ARF6* expression does not affect shoot apical meristem development of wild type. (DOC 43 kb) Additional file 7: Table S5. Reduction of *ARF6* expression does not affect *zll-1* shoot apical meristem development. (DOC 52 kb) Additional file 8: Table S6. Reduced *ARF6* expression does not affect shoot apical meristem development in wild-type background. (DOC 53 kb) Additional file 9: Table S7. Reduced *ARF2* expression partially suppresses *zll-1* shoot apical meristem defects. (DOC 42 kb) Additional file 10: Table S8. Increased *ARF2* expression enhances the frequency of shoot meristem termination in *zll-1. (DOC 48 kb)*
Additional file 11: Figure S3. Expression of *ARF2* but not *ARF3* and *ARF4* is negatively regulated by *AGO10* and *REV. ARF* mRNA levels in torpedo stage embryos of the indicated genotypes relative to wild type. Transcription levels are normalized to the reference gene *At4g26410*. Significance tested by Student’s *t*-test is indicated. ***p* < 0.01, ****p* < 0.001. All other comparisons did not show a significant difference. (PPT 123 kb) Additional file 12: Figure S4. Upregulation of *pARF2:ntdTomato* expression in *zll-1* embryo is suppressed by *rev-10d.* Percentages of *pARF2:ntdTomato* signal strength in *zll-1* embryos of the indicated genotypes are shown. Chi-square test was used to calculate *p*-values. ***p* < 0.01, ****p* < 0.001. *n* number of analyzed embryos. (PPT 120 kb) Additional file 13: Figure S5. Shoot meristem development is partially restored in *zll-1* by *rev10-d.* (A) The severity of shoot meristem defects in *zll-1* is alleviated by the gain-of-function mutation *rev-10d*. (B) Decreased *pCLV3:GFP-er* expression levels in *zll-1* bent-cotyledon and mature embryos are partially suppressed by *rev-10d*. Genotypes and embryo stages are indicated. Chi-square test was used to calculate *p*-values. ****p* < 0.001. *n* number of analyzed embryos. Shoot meristem defects: *empty apex* differentiated stem cell, no organ formation; *pin* single filamentous structure; *1 L* one central leaf; *2 L* termination into two leaves; *wt* wild-type-like. (PPT 162 kb)

## Abbreviations

AFB1–3: AUXIN SIGNALING F-BOX1 auxin receptor 1–3
AGO10: ARGONAUTE 10
amiR: artificial miRNA
ARF2: AUXIN RESPONSE FACTOR 2
ARF2m: mutated ARF2
CLV3: CLAVATA 3
GFP: green fluorescent protein
L*er*: Landsberg *erecta*
qRT-PCR: quantitative reverse transcriptase polymerase chain reaction
REV: REVOLUTA
TAA1: TRYPTOPHAN AMINOTRANSFERASE OF ARABIDOPSIS 1
TAR1/2: TRYPTOPHAN AMINOTRANSFERASE RELATED 1/2
TIR1: TRANSPORT INHIBITOR RESPONSE1
YUC1–4: YUCCA 1–4
ZLL: ZWILLE
