# Supplementary material for: ZLL/AGO10 maintains shoot meristem stem cells during Arabidopsis embryogenesis by down-regulating ARF2-mediated auxin response
Source: BMC Biol. 2015 Sep 10;13:74. doi: 10.1186/s12915-015-0180-y (PMC4565019; doi:10.1186/s12915-015-0180-y)
Supplement: Additional file 1: Figure S1. — DR5:GFP signal is upregulated in zll-1 embryos. Integrated fluorescence intensity of DR5:GFP signal of whole embryos at the indicated stages. n numbers of embryos analyzed, n.s. not significant; ***p < 0.001. (PPT 107 kb) [file 12915_2015_180_MOESM1_ESM.ppt]

## Slide 1
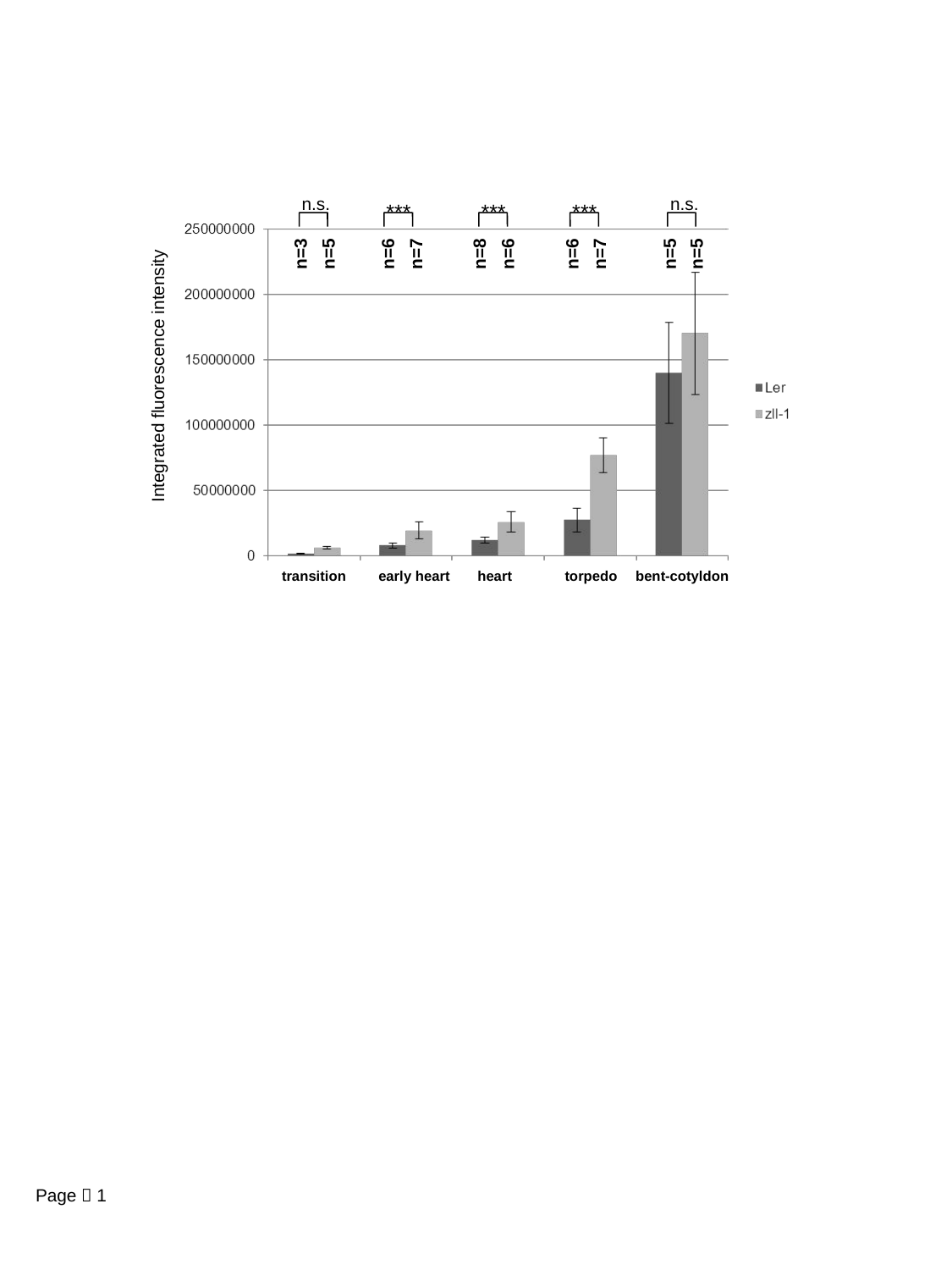

n.s.
n.s.
***
***
***
n=3
n=5
n=6
n=7
n=8
n=6
n=6
n=7
n=5
n=5
Integrated fluorescence intensity
transition
early heart
heart
torpedo
bent-cotyldon
