# Supplementary material for: ZLL/AGO10 maintains shoot meristem stem cells during Arabidopsis embryogenesis by down-regulating ARF2-mediated auxin response
Source: BMC Biol. 2015 Sep 10;13:74. doi: 10.1186/s12915-015-0180-y (PMC4565019; doi:10.1186/s12915-015-0180-y)
Supplement: Additional file 2: Table S1. — Increased auxin response in the zll-1 mutant. (DOC 51 kb) [file 12915_2015_180_MOESM2_ESM.doc]

**Additional file 2: Table S1: Increased auxin response in the *zll-1* mutant**

| **Embryo stage** | **DR5:GFP signal** | ***zll-1*** | **L*er*** |
| --- | --- | --- | --- |
| transition/heart stage |  |  |  |
|  | no signal (%) | 76.9 | 90.0 |
|  | hypophysis (%) | 23.1 | 10.0 |
|  | total (n) | 26 | 50 |
| heart |  |  |  |
|  | hypophysis (%) | 0.0 | 82.9 |
|  | cotyledon + hypophysis (%) | 38.5 | 17.1 |
|  | cotyledon + hypophysis + provasculature (%) | 61.5 | 0.0 |
|  | total (n) | 26 | 50 |
| early torpedo |  |  |  |
|  | hypophysis (%) | 0.0 | 58.2 |
|  | cotyledon & hypophysis (%) | 40.0 | 37.3 |
|  | cotyledon + hypophysis + provasculature (%) | 60.0 | 4.5 |
|  | total (n) | 30 | 67 |
| late torpedo |  |  |  |
|  | hypophysis (%) | 0.0 | 30.9 |
|  | cotyledon & hypophysis (%) | 2.4 | 14.5 |
|  | cotyledon + hypophysis + provasculature (%) | 97.6 | 54.5 |
|  | total (n) | 41 | 55 |
| bent-cotyledon |  |  |  |
|  | cotyledon + hypophysis + provasculature (%) | 100.0 | 100.0 |
|  | total (n) | 35 | 64 |
| *DR5:nlsGFP* expression in L*er* and *zll-1* sibling lines during embryogenesis. Embryo stages and genotypes are indicated. "cotyledon", signal in cotyledonary tips. | | | |
