# Supplementary material for: ZLL/AGO10 maintains shoot meristem stem cells during Arabidopsis embryogenesis by down-regulating ARF2-mediated auxin response
Source: BMC Biol. 2015 Sep 10;13:74. doi: 10.1186/s12915-015-0180-y (PMC4565019; doi:10.1186/s12915-015-0180-y)
Supplement: Additional file 3: Table S2. — DII-Venus levels are reduced in zll-1 mutants. (DOC 65 kb) [file 12915_2015_180_MOESM3_ESM.doc]

**Additional file 3: Table S2: DII-Venus levels are reduced in *zll-1* mutants**

|  | **expression patterns** | | **wild-type** | | | ***zll-1* mutant** | | |
| --- | --- | --- | --- | --- | --- | --- | --- | --- |
| **Embryo stages and numbers (n)** | ***pRPS5a:mDII-ntdTomato*** | ***pRPS5a:DII- n3xVenus*** | **L*er*#1** | **L*er*#2** | **L*er*#3** | ***zll-1*#1** | ***zll-1*#2** | ***zll-1*#3** |
| transition to heart | apical+weak basal | no signal | 0 | 4.3 | 13.3 | 94.4 | 90.5 | 100 |
|  | apical+weak basal | adaxial cot. | 28 | 17.4 | 40 | 5.6 | 0 | 0 |
|  | apical+weak basal | apical+weak basal | 72 | 78.3 | 46.7 | 0 | 9.5 | 0 |
| total (n) |  |  | 25 | 23 | 15 | 18 | 21 | 17 |
|  | | | | | | | | |
| heart stage | apical+weak basal | no signal | 6.7 | 10 | 5.9 | 95.8 | 100 | 100 |
|  | apical+weak basal | Adaxial cot. | 0 | 40 | 29.4 | 4.2 | 0 | 0 |
|  | apical+weak basal | apical+weak basal | 93.3 | 50 | 64.7 | 0 | 0 | 0 |
| total (n) |  |  | 15 | 20 | 17 | 24 | 17 | 16 |
|  | | | | | | | | |
| early torpedo | apical+weak basal | no signal | 31.1 | 0 | 7.1 | 100 | 100 | 100 |
|  | apical+weak basal | adaxial cot. | 44.8 | 81.8 | 28.6 | 0 | 0 | 0 |
|  | apical+weak basal | apical+weak basal | 24.1 | 18.2 | 64.3 | 0 | 0 | 0 |
| total (n) |  |  | 29 | 11 | 14 | 24 | 14 | 18 |
|  | | | | | | | | |
| late torpedo | apical+weak basal | no signal | 16.7 | 8.3 | 0 | 95 | 100 | 100 |
|  | apical+weak basal | apical+very weak basal | 83.3 | 91.7 | 100 | 5 | 0 | 0 |
| total (n) |  |  | 24 | 12 | 34 | 20 | 17 | 20 |
|  | | | | | | | | |
| bent-cotyledon | close to background cot + root | no signal | 100 | 100 | 75.8 | 100 | 92.6 | 90.9 |
|  | close to background cot + root | Close to background cot | 0 | 0 | 24.2 | 0 | 7.4 | 9.1 |
| | total (n) | | --- | |  |  | 20 | 22 | 33 | 19 | 27 | 22 |
| Expression patterns of *pRPS5amDII-ntdTomato* and *pRPS5a:DII-Venus* in three independent lines each of L*er* and *zll-1.* cot; cotyledons. Frequencies are given in %. cot, cotyledons. | | | | | | | | |
