# Supplementary material for: ZLL/AGO10 maintains shoot meristem stem cells during Arabidopsis embryogenesis by down-regulating ARF2-mediated auxin response
Source: BMC Biol. 2015 Sep 10;13:74. doi: 10.1186/s12915-015-0180-y (PMC4565019; doi:10.1186/s12915-015-0180-y)
Supplement: Additional file 4: Figure S2. — Transcript levels of auxin biosynthesis genes TAA1 and TAR2. TAA1 (A) and TAR2 (B) transcript levels are not significantly altered between zll-1 and Ler wild-type embryos. qRT-PCR transcript levels relative to reference gene At4g26410 and standard deviation of three biological replicates are shown. Student’s t-test was used to calculate p-values. (PPT 112 kb) [file 12915_2015_180_MOESM4_ESM.ppt]

## Slide 1
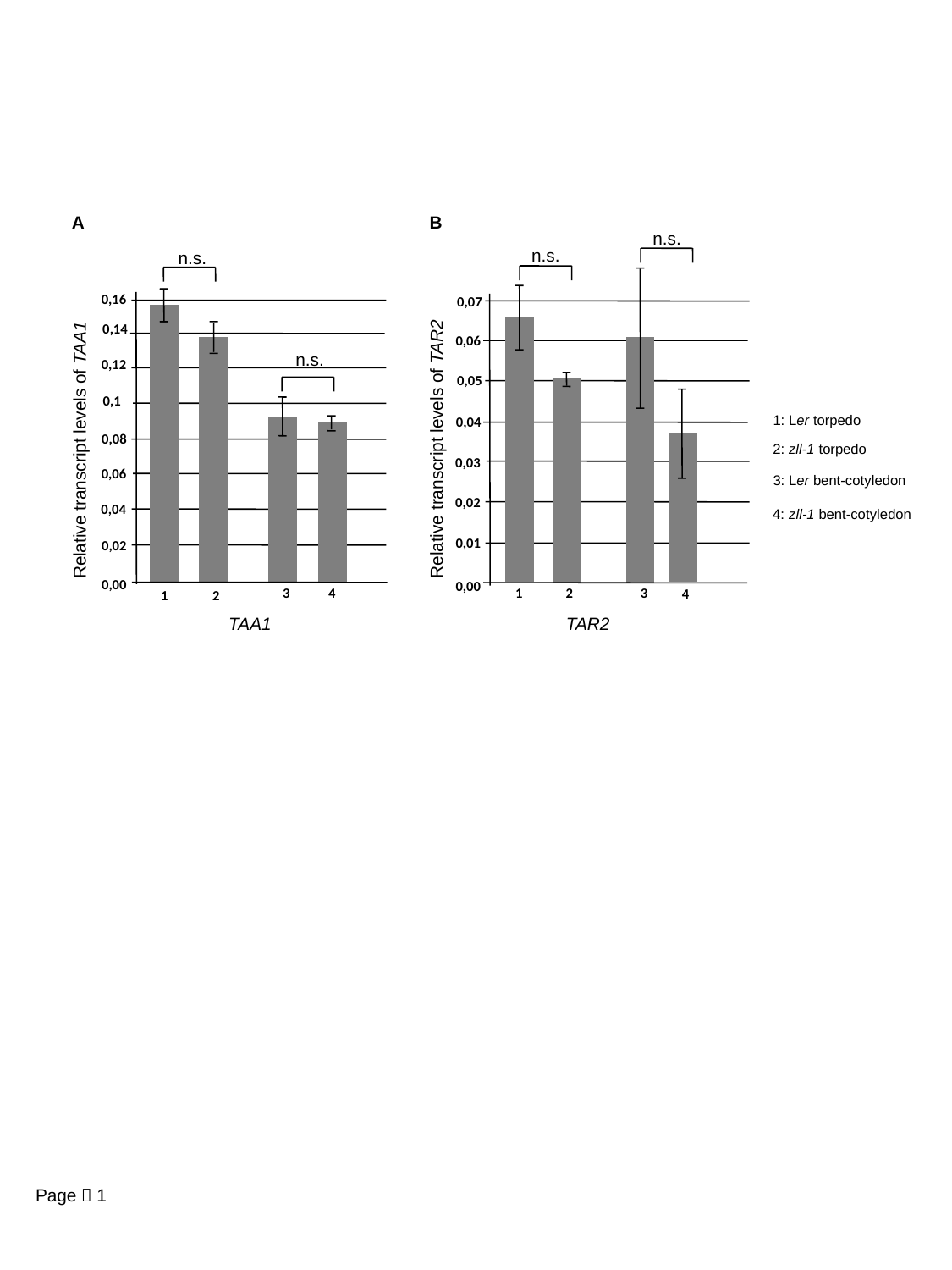

A
B
n.s.
n.s.
n.s.
0,16
0,07
0,14
0,06
n.s.
0,12
0,05
0,1
1: Ler torpedo
0,04
0,08
Relative transcript levels of TAR2
Relative transcript levels of TAA1
2: zll-1 torpedo
0,03
0,06
3: Ler bent-cotyledon
0,02
0,04
4: zll-1 bent-cotyledon
0,01
0,02
0,00
0,00
1
3
4
2
3
4
1
2
TAA1
TAR2
