# Supplementary material for: ZLL/AGO10 maintains shoot meristem stem cells during Arabidopsis embryogenesis by down-regulating ARF2-mediated auxin response
Source: BMC Biol. 2015 Sep 10;13:74. doi: 10.1186/s12915-015-0180-y (PMC4565019; doi:10.1186/s12915-015-0180-y)
Supplement: Additional file 5: Table S3. — Increased ARF6 expression does not affect shoot apical meristem development in zll-1. (DOC 43 kb) [file 12915_2015_180_MOESM5_ESM.doc]

**Additional file 5 Table S3: Increased *ARF6* expression does not affect shoot apical meristem development in *zll-1***

| **Line** | **%SAM defect** | **Total** | **Genotype** |
| --- | --- | --- | --- |
| #1 | 80.1 | 151 | *pRPS5a: ARF6* in *zll-1* |
| #2 | 82.6 | 115 | *pRPS5a: ARF6* in *zll-1* |
| #3 | 79.8 | 84 | *pRPS5a: ARF6* in *zll-1* |
| #4 | 81.8 | 66 | *pRPS5a: ARF6* in *zll-1* |
| #5 | 78.3 | 92 | *pRPS5a: ARF6* in *zll-1* |
| #6 | 79.0 | 176 | *pRPS5a: ARF6* in *zll-1* |
| #7 | 81.6 | 87 | *pRPS5a: ARF6* in *zll-1* |
| #10 | 79.7 | 79 | *pRPS5a: ARF6* in *zll-1* |
| Control* | 80.9 | 110 | *zll-1* |
| ARF6 is driven by pRPS5a. Non-transformant *zll-1* mutant was used as negative control. SAM phenotype was analysed on 14 days old seedlings. * Non transformed *zll-1* | | | |
