# Supplementary material for: ZLL/AGO10 maintains shoot meristem stem cells during Arabidopsis embryogenesis by down-regulating ARF2-mediated auxin response
Source: BMC Biol. 2015 Sep 10;13:74. doi: 10.1186/s12915-015-0180-y (PMC4565019; doi:10.1186/s12915-015-0180-y)
Supplement: Additional file 6: Table S4. — Increased ARF6 expression does not affect shoot apical meristem development of wild type. (DOC 43 kb) [file 12915_2015_180_MOESM6_ESM.doc]

**Additional file 6 Table S4: Increased *ARF6* expression does not affect shoot apical meristem development of wild-type**

| **Line** | **%SAM defect** | **Total** | **Genotype** |
| --- | --- | --- | --- |
| #1 | 0,0 | 210 | *pRPS5a: ARF6* in wild type |
| #2 | 0,0 | 263 | *pRPS5a: ARF6* in wild type |
| #3 | 0,0 | 196 | *pRPS5a: ARF6* in wild type |
| #4 | 0,0 | 287 | *pRPS5a: ARF6* in wild type |
| #5 | 0,0 | 142 | *pRPS5a: ARF6* in wild type |
| #6 | 0,0 | 210 | *pRPS5a: ARF6* in wild type |
| #7 | 0,0 | 178 | *pRPS5a: ARF6* in wild type |
| Control* | 0,0 | 230 | Wild type |
| Non-transformed wild type mutant was used as control. SAM phenotype was analysed on 14 days old seedlings. * Non transformed wild type | | | |
