# Supplementary material for: ZLL/AGO10 maintains shoot meristem stem cells during Arabidopsis embryogenesis by down-regulating ARF2-mediated auxin response
Source: BMC Biol. 2015 Sep 10;13:74. doi: 10.1186/s12915-015-0180-y (PMC4565019; doi:10.1186/s12915-015-0180-y)
Supplement: Additional file 7: Table S5. — Reduction of ARF6 expression does not affect zll-1 shoot apical meristem development. (DOC 52 kb) [file 12915_2015_180_MOESM7_ESM.doc]

**Additional file 7 Table S5: Reduction of *ARF6* expression does not affect *zll-1* shoot apical meristem development**

| **line** | **%SAM defect** | **Total** | **Genotype** |
| --- | --- | --- | --- |
| *#*1 | 21,1 | 250 | *p35S:amiR-ARF6 in zll-1* |
| *#*2 | 19,5 | 190 | *p35S:amiR-ARF6 in zll-1* |
| *#*3 | 22,0 | 186 | *p35S:amiR-ARF6 in zll-1* |
| *#*4 | 23,8 | 193 | *p35S:amiR-ARF6 in zll-1* |
| *#*5 | 25,0 | 204 | *p35S:amiR-ARF6 in zll-1* |
| *#*6 | 24,9 | 245 | *p35S:amiR-ARF6 in zll-1* |
| *#*7 | 27,2 | 305 | *p35S:amiR-ARF6 in zll-1* |
| *#*8 | 22,1 | 412 | *p35S:amiR-ARF6 in zll-1* |
| *#*9 | 22,7 | 198 | *p35S:amiR-ARF6 in zll-1* |
| #10 | 28,4 | 208 | *p35S:amiR-ARF6 in zll-1* |
| #11 | 21,0 | 423 | *p35S:amiR-ARF6 in zll-1* |
| #12 | 23,3 | 417 | *p35S:amiR-ARF6 in zll-1* |
| #13 | 24,9 | 112 | *p35S:amiR-ARF6 in zll-1* |
| #14 | 21,3 | 450 | *p35S:amiR-ARF6 in zll-1* |
| #15 | 25,6 | 432 | *p35S:amiR-ARF6 in zll-1* |
| #16 | 29,8 | 395 | *p35S:amiR-ARF6 in zll-1* |
| #17 | 27,0 | 289 | *p35S:amiR-ARF6 in zll-1* |
| #18 | 26,9 | 256 | *p35S:amiR-ARF6 in zll-1* |
| #19 | 28,9 | 346 | *p35S:amiR-ARF6 in zll-1* |
| #20 | 25,6 | 280 | *p35S:amiR-ARF6 in zll-1* |
| Control* | 22,6 | 451 | *zll-1* |
| Frequencies of defective shoot meristems (SAM) in 14-day-old *zll-1* *amiR-arf6* seedlings. Line numbers represent independent transformants. *Non-transformed *zll-1*. n, total number of seedlings analyzed. | | | |
