# Supplementary material for: ZLL/AGO10 maintains shoot meristem stem cells during Arabidopsis embryogenesis by down-regulating ARF2-mediated auxin response
Source: BMC Biol. 2015 Sep 10;13:74. doi: 10.1186/s12915-015-0180-y (PMC4565019; doi:10.1186/s12915-015-0180-y)
Supplement: Additional file 8: Table S6. — Reduced ARF6 expression does not affect shoot apical meristem development in wild-type background. (DOC 53 kb) [file 12915_2015_180_MOESM8_ESM.doc]

**Additional file 8 Table S6: Reduced *ARF6* expression does not affect shoot apical meristem development in wild-type background**

| **line** | **%SAM defect** | **Total** | **Genotype** |
| --- | --- | --- | --- |
| *#*1 | 0,00 | 130 | *p35S:amiR-ARF6 in* wt |
| *#*2 | 0,00 | 145 | *p35S:amiR-ARF6 in* wt |
| *#*3 | 0,00 | 135 | *p35S:amiR-ARF6 in* wt |
| *#*4 | 0,00 | 250 | *p35S:amiR-ARF6 in* wt |
| *#*5 | 0,00 | 351 | *p35S:amiR-ARF6 in* wt |
| *#*6 | 0,00 | 243 | *p35S:amiR-ARF6 in* wt |
| *#*7 | 0,00 | 198 | *p35S:amiR-ARF6 in* wt |
| *#*8 | 0,00 | 176 | *p35S:amiR-ARF6 in* wt |
| *#*9 | 0,00 | 168 | *p35S:amiR-ARF6 in* wt |
| *#*10 | 0,00 | 179 | *p35S:amiR-ARF6 in* wt |
| *#*11 | 0,00 | 207 | *p35S:amiR-ARF6 in* wt |
| *#*12 | 0,00 | 193 | *p35S:amiR-ARF6 in* wt |
| *#*13 | 0,00 | 301 | *p35S:amiR-ARF6 in* wt |
| *#*14 | 0,00 | 297 | *p35S:amiR-ARF6 in* wt |
| *#*15 | 0,00 | 285 | *p35S:amiR-ARF6 in* wt |
| Control* | 0,00 | 200 | wt |
| Frequencies of defective shoot meristems (SAM) in 14-day-old *amiR-arf6* seedlings. Line numbers represent independent transformants. *Non-transformed wild type. n, total number of seedlings analyzed. | | | |
