# Supplementary material for: ZLL/AGO10 maintains shoot meristem stem cells during Arabidopsis embryogenesis by down-regulating ARF2-mediated auxin response
Source: BMC Biol. 2015 Sep 10;13:74. doi: 10.1186/s12915-015-0180-y (PMC4565019; doi:10.1186/s12915-015-0180-y)
Supplement: Additional file 9: Table S7. — Reduced ARF2 expression partially suppresses zll-1 shoot apical meristem defects. (DOC 42 kb) [file 12915_2015_180_MOESM9_ESM.doc]

**Additional file 9 Table S7: Reduced *ARF2* expression partially suppresses *zll-1* shoot apical meristem defects**

| **Line** | **Defective SAM (%)** | **n** | **Genotype** |
| --- | --- | --- | --- |
| #1 | 52.4 | 267 | *p35S:amiR-arf2 in zll-1* |
| #2 | 57.5 | 140 | *p35S:amiR-arf2 in zll-1* |
| #3 | 52.3 | 132 | *p35S:amiR-arf2 in zll-1* |
| #4 | 53.3 | 45 | *p35S:amiR-arf2 in zll-1* |
| #5 | 37.7 | 61 | *p35S:amiR-arf2 in zll-1* |
| control | 0 | 451 | *p35S:amiR-arf2 in* L*er* |
| *zll-1** | 83.5 | 115 | *zll-1* |
| Frequencies of defective shoot meristems (SAM) in 14-day-old *zll-1* and *zll-1amiR-arf2* seedlings. Line numbers represent independent transformants. *zll-1** carries a control construct. n, total number of seedlings analyzed. | | | |
