# Supplementary material for: ZLL/AGO10 maintains shoot meristem stem cells during Arabidopsis embryogenesis by down-regulating ARF2-mediated auxin response
Source: BMC Biol. 2015 Sep 10;13:74. doi: 10.1186/s12915-015-0180-y (PMC4565019; doi:10.1186/s12915-015-0180-y)
Supplement: Additional file 10: Table S8. — Increased ARF2 expression enhances the frequency of shoot meristem termination in zll-1. (DOC 48 kb) [file 12915_2015_180_MOESM10_ESM.doc]

**Additional file 10 Table S8: Increased *ARF2* expression enhances the frequency of shoot meristem termination in *zll-1***

| **Line** | **Defective SAM (%)** | **n** | **Genotype** |
| --- | --- | --- | --- |
| #1 | 92.0 | 226 | *pARF2:mARF2* in *zll-1* |
| #3 | 97.0 | 132 | *pARF2:mARF2* in *zll-1* |
| #5 | 92.7 | 12 | *pARF2:mARF2* in *zll-1* |
| #15 | 88.1 | 159 | *pARF2:mARF2* in *zll-1* |
| #17 | 88.6 | 271 | *pARF2:mARF2* in *zll-1* |
| #23 | 96.5 | 57 | *pARF2:mARF2* in *zll-1* |
| #26 | 89.3 | 28 | *pARF2:mARF2* in *zll-1* |
| #25 | 90.9 | 88 | *pARF2:mARF2* in *zll-1* |
| #24 | 96.8 | 93 | *pARF2:mARF2* in *zll-1* |
| #11 | 96.3 | 109 | *pARF2:mARF2* in *zll-1* |
| #14 | 94.4 | 248 | *pARF2:mARF2* in *zll-1* |
| *zll-1* | 77.5 | 111 | *zll-1* |
| Expression of the *TAS3*-resistant *ARF2* from its native promoter increases frequencies of terminated shoot meristems in *zll-1*. 14-days-old seedlings are analyzed. n, total number of seedlings. | | | |
