# Supplementary material for: ZLL/AGO10 maintains shoot meristem stem cells during Arabidopsis embryogenesis by down-regulating ARF2-mediated auxin response
Source: BMC Biol. 2015 Sep 10;13:74. doi: 10.1186/s12915-015-0180-y (PMC4565019; doi:10.1186/s12915-015-0180-y)
Supplement: Additional file 11: Figure S3. — Expression of ARF2 but not ARF3 and ARF4 is negatively regulated by AGO10 and REV. ARF mRNA levels in torpedo stage embryos of the indicated genotypes relative to wild type. Transcription levels are normalized to the reference gene At4g26410. Significance tested by Student’s t-test is indicated. **p < 0.01, ***p < 0.001. All other comparisons did not show a significant difference. (PPT 123 kb) [file 12915_2015_180_MOESM11_ESM.ppt]

## Slide 1
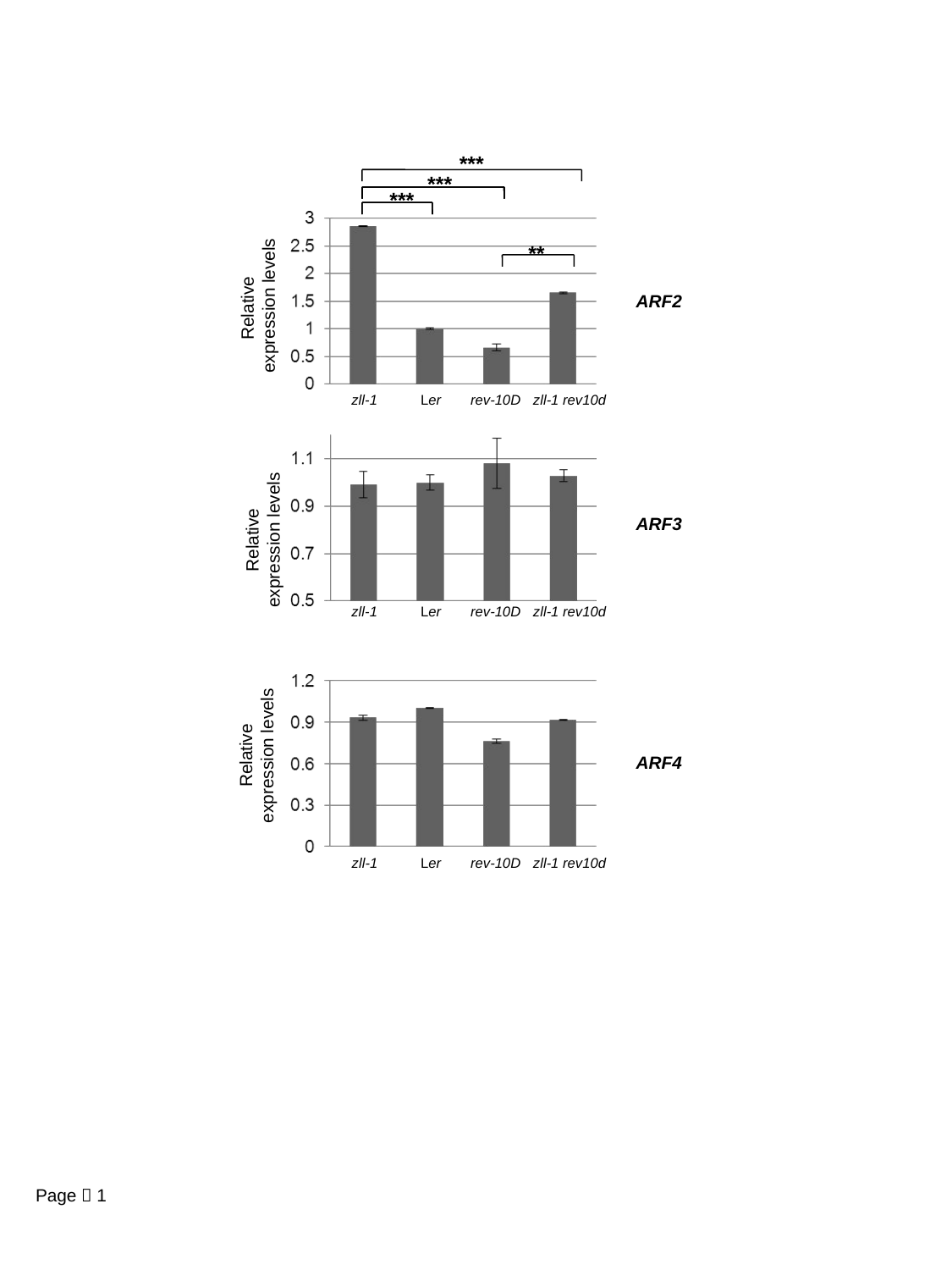

***
***
***
**
Relative
expression levels
ARF2
zll-1
Ler
rev-10D
zll-1 rev10d
ARF3
Relative
expression levels
zll-1
Ler
rev-10D
zll-1 rev10d
Relative
expression levels
ARF4
zll-1
Ler
rev-10D
zll-1 rev10d
