# Supplementary material for: ZLL/AGO10 maintains shoot meristem stem cells during Arabidopsis embryogenesis by down-regulating ARF2-mediated auxin response
Source: BMC Biol. 2015 Sep 10;13:74. doi: 10.1186/s12915-015-0180-y (PMC4565019; doi:10.1186/s12915-015-0180-y)
Supplement: Additional file 12: Figure S4. — Upregulation of pARF2:ntdTomato expression in zll-1 embryo is suppressed by rev-10d. Percentages of pARF2:ntdTomato signal strength in zll-1 embryos of the indicated genotypes are shown. Chi-square test was used to calculate p-values. **p < 0.01, ***p < 0.001. n number of analyzed embryos. (PPT 120 kb) [file 12915_2015_180_MOESM12_ESM.ppt]

## Slide 1
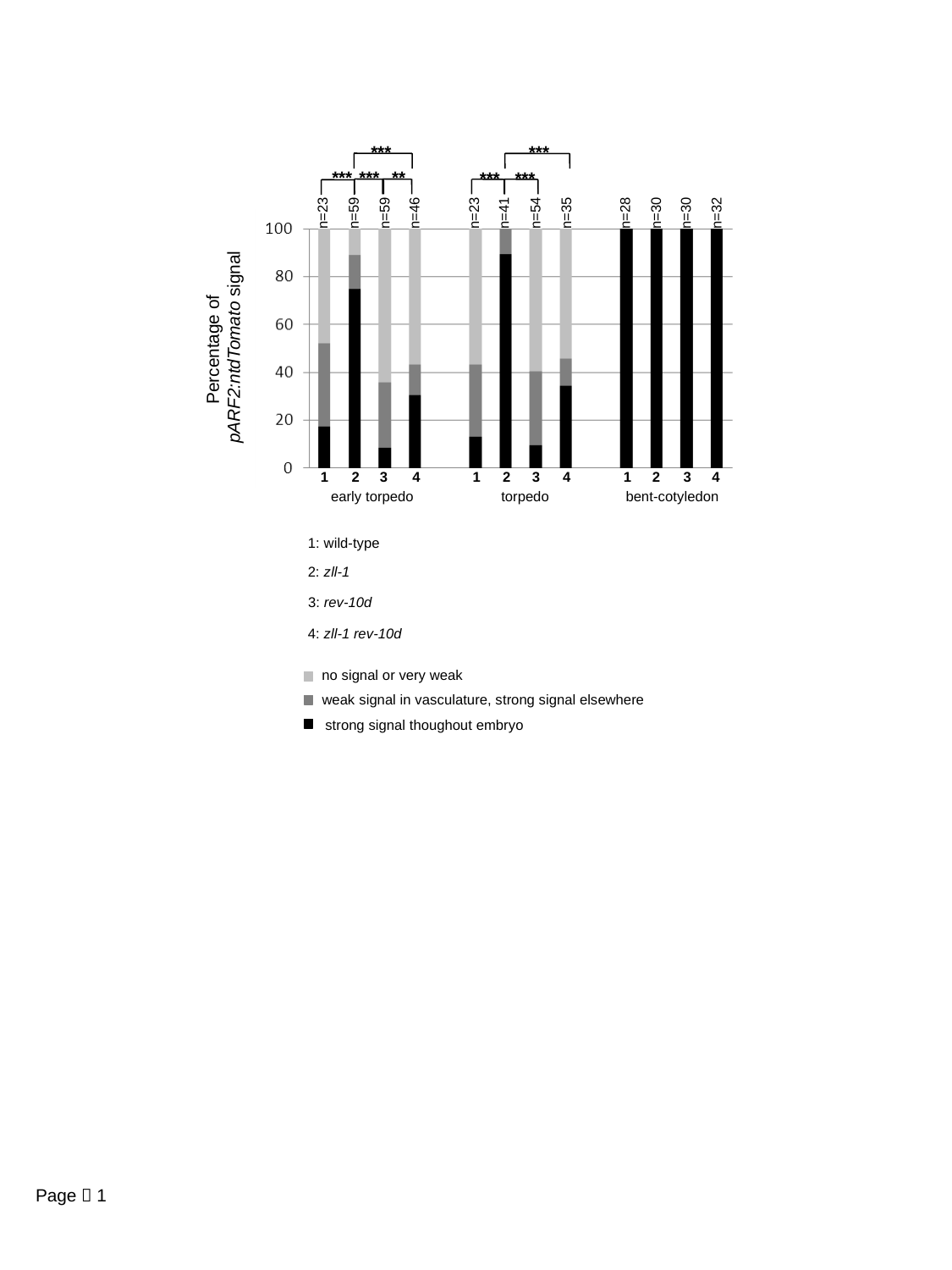

***
***
***
***
**
***
***
n=23
n=59
n=59
n=46
n=23
n=41
n=54
n=35
n=28
n=30
n=30
n=32
Percentage of
pARF2:ntdTomato signal
1
2
3
4
1
2
3
4
1
2
3
4
early torpedo
torpedo
bent-cotyledon
1: wild-type
2: zll-1
3: rev-10d
4: zll-1 rev-10d
no signal or very weak
weak signal in vasculature, strong signal elsewhere
strong signal thoughout embryo
