# Supplementary material for: ZLL/AGO10 maintains shoot meristem stem cells during Arabidopsis embryogenesis by down-regulating ARF2-mediated auxin response
Source: BMC Biol. 2015 Sep 10;13:74. doi: 10.1186/s12915-015-0180-y (PMC4565019; doi:10.1186/s12915-015-0180-y)
Supplement: Additional file 13: Figure S5. — Shoot meristem development is partially restored in zll-1 by rev10-d. (A) The severity of shoot meristem defects in zll-1 is alleviated by the gain-of-function mutation rev-10d. (B) Decreased pCLV3:GFP-er expression levels in zll-1 bent-cotyledon and mature embryos are partially suppressed by rev-10d. Genotypes and embryo stages are indicated. Chi-square test was used to calculate p-values. ***p < 0.001. n number of analyzed embryos. Shoot meristem defects: empty apex differentiated stem cell, no organ formation; pin single filamentous structure; 1 L one central leaf; 2 L termination into two leaves; wt wild-type-like. (PPT 162 kb) [file 12915_2015_180_MOESM13_ESM.ppt]

## Slide 1
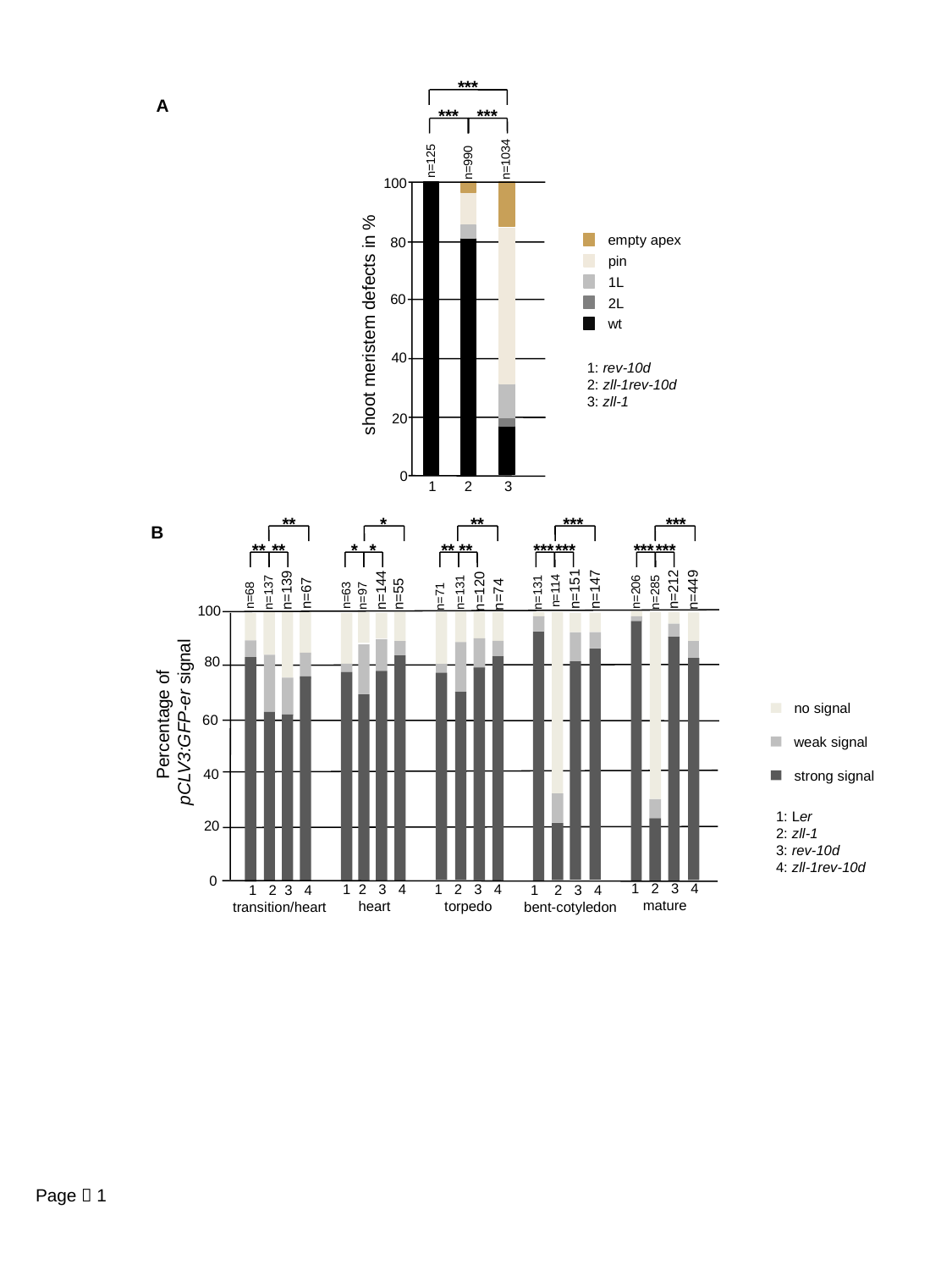

***
A
***
***
n=1034
n=125
n=990
100
empty apex
80
pin
1L
60
2L
shoot meristem defects in %
wt
40
1: rev-10d
2: zll-1rev-10d
3: zll-1
20
0
1 2 3
**
*
**
***
***
B
**
**
*
*
**
**
***
***
***
***
n=151
n=147
n=67
n=212
n=449
n=55
n=144
n=139
n=74
n=120
n=114
n=206
n=137
n=131
n=131
n=285
n=68
n=63
n=97
n=71
100
80
no signal
weak signal
strong signal
Percentage of
pCLV3:GFP-er signal
60
40
1: Ler
2: zll-1
3: rev-10d
4: zll-1rev-10d
20
0
1 2 3 4
mature
1 2 3 4
 heart
 1 2 3 4
 torpedo
 1 2 3 4
 transition/heart
1 2 3 4
 bent-cotyledon
